# Combustion derived single phase Y_4_Al_2_O_9_:Tb^3+^ nanophosphor: crystal chemistry and optical analysis for solid state lighting applications†

**DOI:** 10.1039/d3ra00735a

**Published:** 2023-03-08

**Authors:** Pawan Kumar, Devender Singh, Isha Gupta, Sitender Singh, Simran Nehra, Ramesh Kumar

**Affiliations:** a Department of Chemistry, Maharshi Dayanand University Rohtak-124001 Haryana India devjakhar@gmail.com; b CSIR-National Physical Laboratory Dr K. S. Krishnan Marg New Delhi-110012 India; c Department of Chemistry, Kurukshetra University Kurukshetra-136119 Haryana India

## Abstract

Cool green light emanating monoclinic Y_4−*x*_Al_2_O_9_:*x*Tb^3+^ (*x* = 1–5 mol%) nanophosphors have been fabricated through gel-combustion method. X-ray diffraction and transmission electron-microscopy data have been utilized to assess their structural and microstructural characteristics, including cell parameters and crystallite size. Uneven aggregation of nanoparticles in the nano-scale with distinctive porosity can be seen in the TEM micrograph. Kubelka–Munk model imitative diffuse reflectance spectra and an optical band gap of 5.67 eV for the Y_3.97_Al_2_O_9_:0.03Tb^3+^ nanophosphor revealed high optical quality in the samples, which were thought to be non-conducting. The emission (PL) and excitation (PLE) spectra as well as lifetime measurements have been used to determine the luminescence characteristics of the synthesized nanophosphors. The emission spectra show two color *i.e.* blue color due to ^5^D_3_ → ^7^F_*J*_ (*J* = 4 and 5) transitions and green color due to ^5^D_4_ → ^7^F_*J*_ (*J* = 3, 4, 5 and 6) transitions. The most dominant transition (^5^D_4_ → ^7^F_5_) at 548 nm was responsible for the greenish color in focused nanocrystalline samples. Calculated colorimetric characteristics such as CIE, and CCT along with color purity of the synthesized nanocrystalline materials make them the best candidate for the solid-state lighting (SSL).

## Abstract 2

Cool green light emanating monoclinic Y_4−*x*_Al_2_O_9_:*x*Tb^3+^ (*x* = 1–5 mol%) nanophosphors have been fabricated through gel-combustion method.

## Graphical abstract

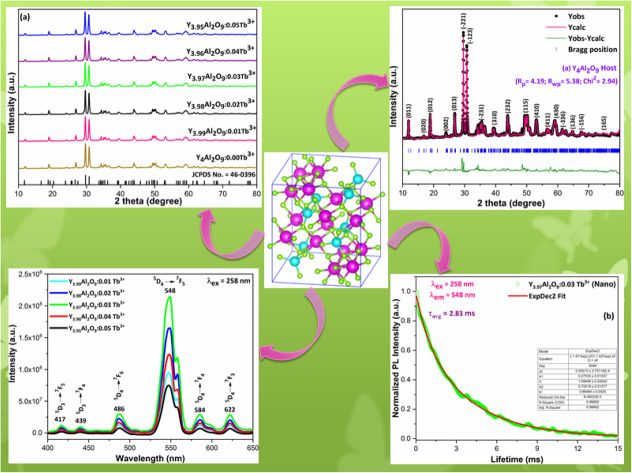

## 1 Introduction

Development of the upcoming generation of highly power efficient, sustainably manufactured solid state lighting (SSL) devices is a primary concern for scientists and researchers globally, especially material scientists, as the world is now experiencing an energy crisis.^1–5^ The significant quantity of power wasted in our modern world is due to artificial lights. Solid state illumination technology has completely revolutionized the field of luminance due to its exceptional properties, which include high energy efficacy, superior optical assets, excellent lumen performance, and environmental stewardship.^6–12^ It is designed to meet the global demand for both indoor and outdoor lighting. Moreover, host matrixes based on aluminates that have enhanced luminance efficiency, excellent CCT and CRI (color rendering index), superior crystallinity, simple synthesis, superior mechanical-strength, significantly greater chemical and thermal stability, temperature resistance, *etc.*, are extremely desirable for doping with an appropriate dopant ion.^13–20^ For solid state lighting and display, yttrium and aluminium host nanophosphors are particularly beneficial. Yttrium aluminium perovskite (Y/Al; 1 : 1) is utilised as a scintillator, and rare earth (RE) activated yttrium aluminium garnet (Y/Al; 0.6 : 1) is frequently employed as a host material for laser action.^21–24^ Nevertheless, there have been very few reports of spectroscopic studies on Y_4_Al_2_O_9_ (Y/Al; 2 : 1) doped with rare earth ions (RE^3+^). The crystal structure of Yttrium aluminium oxide (Y_4_Al_2_O_9_) abbreviated as YAM corresponds to monoclinic system with space-group *P*2_1_/*c*.^25^ The Y atoms are coordinated to either six or seven oxygen atoms with site symmetry C1. The Al atoms are present in four coordination environment with oxygen atoms.^26^

Compared to Y_3_Al_5_O_12_ and YAlO_3_ systems, the YAM host has a lattice structure that allows four unique places where trivalent rare earth (RE^3+^) ions might be replaced. Only a few articles have described the spectroscopic characteristics of the RE^3+^ doped YAM crystal revealing its multi-tenant characteristics. Due to the partially filled 4f-subshell in the RE ions, which is shielded by the surrounding 5s and 5p shells, rare-earth (RE) ions function as dopant in phosphors nanocrystalline materials.^27–29^ The remarkable luminous properties of rare earth activated phosphors are caused by the emission (PL) spectra of produced f–f transitions, which typically produce luminous, precise peaks in visible-range (400–800 nm) of spectrum. Trivalent terbium (Tb^3+^) ion is known to be a vivid green emitter among the trivalent lanthanide ions due to its distinctive ^5^D_4_ → ^7^F_5_ transition.^30^ It is a remarkable host activator having great applicability in SSL and display technology. The existing research indicated that the coprecipitation approach, traditional solid state reaction method and Pechini method were often used to generate the rare earth doped Y_4_Al_2_O_9_ nanomaterials.^31–33^ But, these synthetic methods suffer from numerous confines like non-homogeneous nature, necessity of higher-temperature, more consumption of time *etc.*^34^ In context of current study, low temperature based gel combustion (GC) method was employed to synthesized Y_4−*x*_Al_2_O_9_:*x*Tb^3+^ (*x* = 1–5 mol%) crystalline materials and a systematically investigation about their structural and photophysical characteristics have been done. X-ray diffraction was utilised to investigate the crystal structure. The functional groups found in host were examined using Fourier transform-infrared (FTIR) spectroscopy. Morphology of the material is analyzed by TEM study. The comparative percentage of integral atoms in fabricated nanomaterials is studied *via* energy-dispersive X-ray (EDX) exploration. Luminescent parameters revealed that the synthesized materials are appropriate candidate for SSL applications.

## 2 Experimental

### 2.1 Materials used

Bulk materials *i.e.* Y_4_Al_2_O_9_ (YAM) & optimum Y_3.97_Al_2_O_9_:0.03Tb^3+^ and a series of Y_4_Al_2_O_9_:Tb^3+^ nanophosphors with diverse concentration of Tb^3+^ cation (1–5 mol%) have been synthesized *via* solid state reaction and gel-combustion method respectively. For preparation purpose, urea has been utilized as fuel. The chemicals *viz.*, nitrates of yttrium, terbium and aluminium was used for preparation purpose, purchased from Sigma-Aldrich. Deionized water was used as solvent.

### 2.2 Synthesis of bulk materials

To prepare the bulk materials, stoichiometric amounts of initial chemicals were taken without any further purification. All of them were mixed and grinded uniformly for 1 h using mortar & pestle. Grinded mixture was then kept in an alumina crucible and sintered for 4 h at 1100 °C in a box furnace. Finally calcined material was ground thoroughly again to get the resultant fine bulk phosphor for different analysis.

### 2.3 Synthesis of nanophosphors

Firstly, starting materials were taken in desired stoichiometric proportion and dissolved completely in water. Upon heating at ∼80 °C, this prepared mixture was become viscous have gel type appearance due to vaporization of solvent (H_2_O) molecules. Then, a considered quantity of fuel with deionized (DI) water has been added to resultant solution and further allowed to heat. The formed gel type mixture was allowed to combust for fifteen minutes in a pre-heated furnace at 600 °C. In this single step preparation technique, gaseous products *viz.*, oxides of carbon and nitrogen expelled out during combustion.^35,36^ The low reaction temperature is answerable for formation of nano-crystalline materials. This combustion technique is exothermic in nature which results in high crystallinity of synthesized materials. The porous product was allowed to cool with subsequent grinding to obtain fine powdered material. The formed powdered sample was further calcined at 800 °C to obtain desired Y_4_Al_2_O_9_:Tb^3+^ nanophosphors.

### 2.4 Instrumentations

The crystalline phases were examined on a “Rigaku Ultima X-ray diffractometer” (operating potential: 40 kV & current: 40 mA) and mono-chromatic radiations were produced from Cu-anode tube linked with Johansson-monochromator for Kα_1_ (*λ* = 1.54059 Å). The Bragg–Brentano geometry was obtained in range of 2*θ* from 10° to 80° with a step interval of 0.02° and scan pace of 2° min^−1^. For undoped and doped samples, Rietveld analysis was performed to obtain crystallographic and refinement parameters. The vibrational spectra were obtained on “PerkinElmer 5700 FTIR spectrometer” in solid state by using pellets of anhydrous potassium bromide. To analyze the content of constituent elements, the EDX patterns of prepared nano-samples were recorded. To examine the structural analysis was done through transmission electron microscope (JEOL JEM-1400 Plus). For obtaining optical band gap data of phosphors, their respective reflectance spectra were recorded on a UV-3600 Plus, Shimadzu UV-Vis-NIR spectrophotometer (DRS). Photoluminescence spectral profiles and quantum yield were recorded using a Horiba Jobin YVON Fluorolog Model FL-3-11 equipped a 150 W pulsed xenon lamp. The luminescence lifetime has been noted on a Hitachi F-7000 FL spectrophotometer.

## 3 Results and discussion

### 3.1 XRD evaluation

**Fig. 1:**
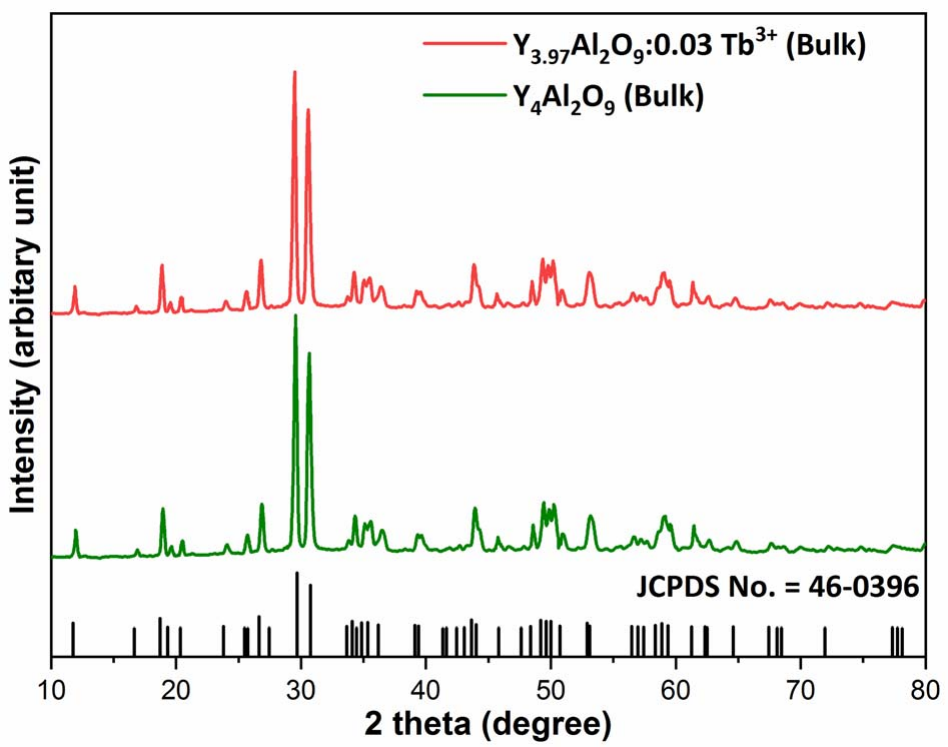
Diffraction patterns of the host and Y_3.97_Al_2_O_9_:0.03Tb^3+^ bulk materials.

**Fig. 2:**
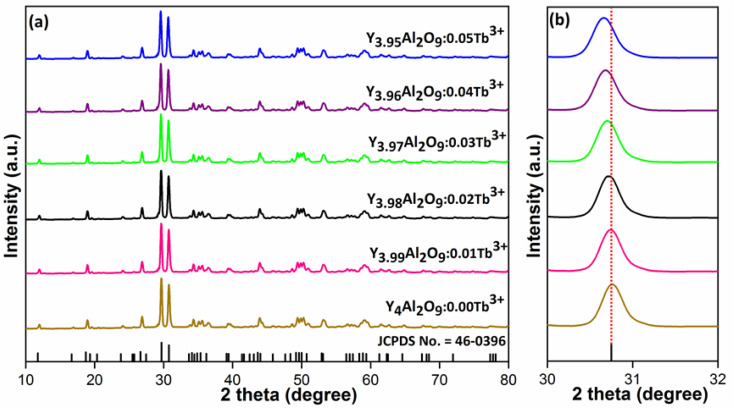
Diffractograms of (a) all synthesized nanophosphors and (b) enlarged XRD patterns.

**Fig. 3:**
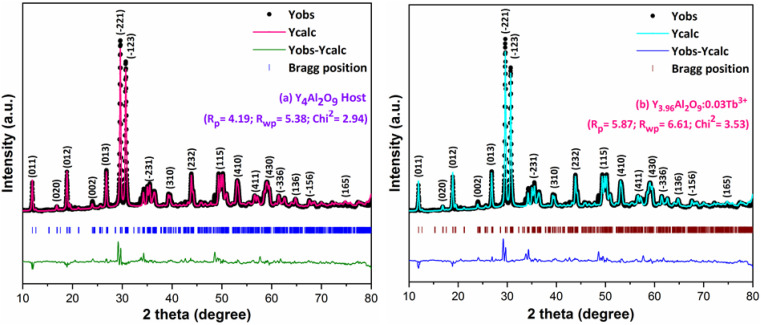
Rietveld profiles for (a) host Y_4_Al_2_O_9_ and (b) Y_3.97_Al_2_O_9_:0.03Tb^3+^ sample.

**Fig. 4:**
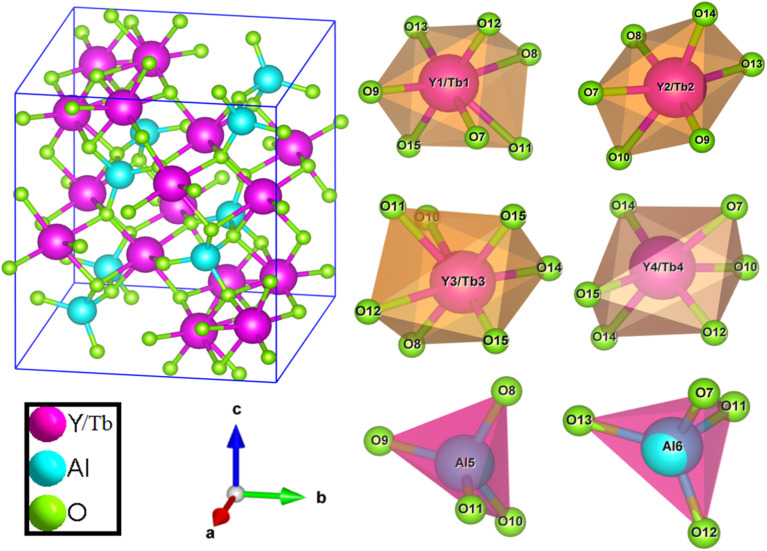
Pictorial representation of crystal structure and atomic environment of each metal atom present in Y_3.97_Al_2_O_9_:0.03Tb^3+^ nanocrystalline material.

**Fig. 5:**
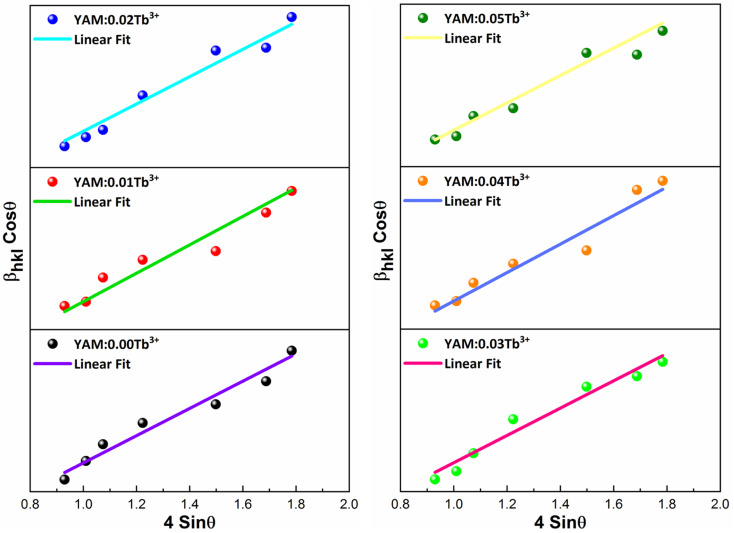
W–H graph of Y_4_Al_2_O_9_ host material and Y_4−*x*_Al_2_O_9_:*x*Tb^3+^ (*x* = 1–5 mol%) samples.

XRD Patterns of synthesized bulk and nanophosphors have been extensively explored to determine crystalline nature and phase identification. Fig. 1 demonstrates the diffraction patterns of bulk materials such as host YAM and Y_3.97_Al_2_O_9_:0.03Tb^3+^ materials. All of the peaks are well matched with the JCPDS card 46-0396.^37^ There is no additional impurity peak in the XRD pattern, confirming single phase of bulk materials and also monoclinic structure having space group *P*2/*c*. High intensity of the XRD peaks defines their high degree of crystallinity as compare to the nanophosphors which was the result of high sintering temperature during the solid state reaction route. The average crystallite size (*D*) of the host and Y_3.97_Al_2_O_9_:0.03Tb^3+^ bulk materials were found to be 127.02 nm and 122.66 nm respectively, which is much higher compared to the nanophosphors. Fig. 2(a) depicts the diffraction spectra of undoped Y_4_Al_2_O_9_ and different concentration (1–5 mol%) of Tb^3+^ doped Y_4_Al_2_O_9_ nanophosphors. All synthetic materials are crystallized, as seen by the diffraction patterns. The diffraction profiles of the prepared nanosamples are well accorded with standard JCPDS number 46-0396,^37^ which affirm existence of monoclinic-crystal phase with *P*2_1_/*c* space-group.^38^ After addition of dopant ion with different concentration in the host materials, there is no additional peaks were noticed in the XRD spectral profiles which confirm the pure synthesis of the Y_4−*x*_Al_2_O_9_:*x*Tb^3+^ (*x* = 1–5 mol%) nanophosphors. However, some alternation in the peaks position is observed in the XRD lines of the doped samples, which was the evidence of Tb^3+^ content in the prepared doped samples. The XRD peaks were shifted in the lower angle side with the incorporation of dopant ion concentration, this was due to replacement of smaller size Y^3+^ ion by the of bigger size Tb^3+^ ion in the host material, as demonstrated in Fig. 2(b). Due to substitution of smaller host ion by larger size activator ion, it was noted that the estimated interspacing *d*-values increased with concentration of activator ions as summarized in the Table 1. It is validate with the Bragg's relation (2*d* sin *θ* = *nλ*) that to remain *nλ* constant, 2*θ*-angle would be decreased, which resembles with the shifting of peaks in the lower angle side.^39^ There are two replicating sites of host material (Y^3+^ and Al^3+^) are available for the activator ion (Tb^3+^) because of their same charge. To assess the viability of replacement, the percentage differences between the dopant and host ions should not be exceed than 30%.^40^ Relation (1) given below is exploited for assessment percentage radii difference between the host and dopant ions.^41^1
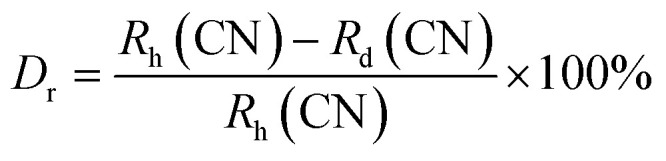
Where, *R*_h_(CN) & *R*_d_(CN) represent ionic-radii of host and incoming cation alongwith coordination number, separately. Computed *D*_r_-value has been found to be less than 30%, which demonstrates successful substitution of Y^3+^ by Tb^3+^ ion. The intensity profile is assessed using the Rietveld refinement method, which enables an approximate model with a real foundation for crystal and refinement characteristics of synthesized materials. Fig. 3(a) & (b) exhibits Rietveld profiles of Y_4_Al_2_O_9_ and Y_3.97_Al_2_O_9_:0.03Tb^3+^ nanophosphor individually. Due to the reliability factors being observed within a sufficient range, all refined data was well established over with initial structural-model. Refinement characteristics of Y_4_Al_2_O_9_ and optimized Y_3.97_Al_2_O_9_:0.03Tb^3+^ nanomaterials are listed in Table 2. Remarkably, all samples are belonging to the monoclinic crystal system with space group *P*2_1_/*c*. From the Table 2, it is clear that the crystallographic parameters including *a*, *b*, *c* and volume of cell increase from host material to optimized sample. This rise in cell parameters further sustains the effective inclusion of larger size Tb^3+^ in host matrix. To better comprehend the structure and inclusion of Tb^3+^ in Y_4_Al_2_O_9_ host, the crystal structure of host material with monoclinic phase is shown in Fig. 4. A unit cell of Y_4_Al_2_O_9_ has 60 atoms, including four Y, two Al, and nine non-equivalent O sites. In the crystal structure of host, 2 Y atoms are in seven coordination number (forming Y–O decahedral unit) and the other 2 Y atoms are 6 coordinated (Y–O octahedron) with oxygen atom. Both the aluminium (Al1 and Al2) atoms are formed tetrahedral unit with four O-atoms.^42,43^ When trivalent terbium ions are doped into YAM host matrix, they replace the position of the Y^3+^ ions host material and making the lattice more distorted. It is obvious that Tb^3+^ ion in Y_4_Al_2_O_9_ host, occupy crystallographically different sites Y1/Tb1, Y3/Tb3, Y2/Tb2 and Y4/Tb4, as displayed in Fig. 4. Every group of emission line of Tb^3+^ contains contribution from different centers. The computed interionic bond distance (Å) between integral atoms of activated nanophosphor is concise in Table S1.† Table S2† itemized the refined atomic-positions and displacement-factors of optimum activated nanophosphor. Additionally, crystallite size of the prepared samples was determined by utilizing Scherer's relation (2).^44^2
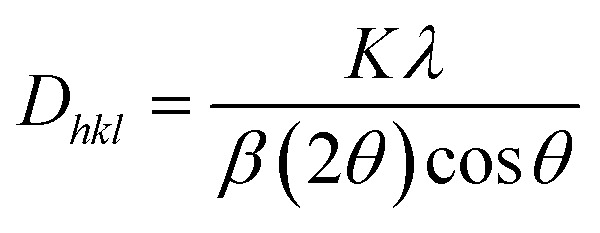
Here, *D*_*hkl*_ symbolizes crystallite size, *λ* used for X-ray wavelength, *β* denotes width of pure diffraction patterns in radians, and *K* becomes constant (0.89). The conceivable estimation of the crystallite size for Y_4_Al_2_O_9_ (YAM) and Y_4−*x*_Al_2_O_9_:*x*Tb^3+^ (*x* = 1–5 mol%) nanomaterials was around 37–42 nm. Also, the existence of microstrain in crystalline material results into peak shifting and line broadening in diffraction patterns. Hence, using W–H linear fitting method, the strain present in the considered nanosamples was assessed by using eqn (3).^45^3
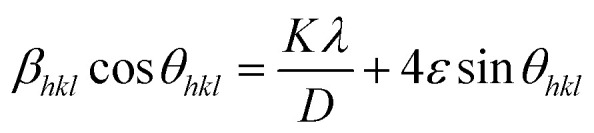
Here, *β*_*hkl*_ is peak broadening and *D* & *λ* are crystallite size and X-rays wavelength respectively. W–H linear fitted graph for undoped YAM and YAM:*x*Tb^3+^ (*x* = 1–5 mol%) in Fig. 5 owing straight line graph between 4 sin *θ*_*hkl*_ (on *x*-axis) and *β*_*hkl*_ cos *θ*_hkl_ (on *y*-axis). The crystallite-size and induced microstrain were determined from values of intercept and slope separately. The computed values are tabulated in Table 3.

**Table 1:** Calculated *d*-spacing values of Y_4_Al_2_O_9_ and Y_4−*x*_Al_2_O_9_:*x*Tb^3+^ (*x* = 1–5 mol%) nanophosphors

Tb^3+^-content (*x*)	2*θ* (−221)	*d* (−221)/Å
0 mol%	30.76	2.9044
1 mol%	30.73	2.9072
2 mol%	30.69	2.9109
3 mol%	30.65	2.9146
4 mol%	30.62	2.9173
5 mol%	30.58	2.9211

**Table 2:** Rietveld refinement outcomes of undoped Y_4_Al_2_O_9_ and optimized Y_3.97_Al_2_O_9_:0.03Tb^3+^ nanophosphors

Sample	Y_4_Al_2_O_9_	Y_3.97_Al_2_O_9_:0.03Tb^3+^
Formula weight	553.47	555.56
Crystal-system	Monoclinic	Monoclinic
Lattice type symmetry	*P*	*P*
Space group	*P*2_1_/*c*	*P*2_1_/*c*
Space group number	14	14
Formula unit (*Z*)	4	4
Pearson symbol	mP60	mP60
*a* (Å)	7.3859	7.4162
*b* (Å)	10.4691	10.5478
*c* (Å)	11.1026	11.1812
*α* = *γ*	90.0000	90.0000
*β*	108.639	108.639
Volume (Å^3^)	813.423	828.725
Density (g cm^−3^)	4.51	4.45
*R*-Factors	4.19, 5.38	5.87, 6.61
*χ* ^2^	2.94	3.53

**Table 3:** Computed XRD parameters *viz.* crystallite size, microstrain, dislocation density of developed YAM and YAM:*x*Tb^3+^ (*x* = 1–5 mol%) nanomaterials

Nanosample	FWHM	Crystallite size (nm)		Microstrain (*ε* × 10^−3^)	Dislocation density (×10^−4^)
		Scherrer's	W–H		
YAM:0.00Tb^3+^	0.1895 ± 0.00589	42.31 ± 1.018	63.16 ± 1.568	3.862 ± 0.0811	5.586
YAM:0.01Tb^3+^	0.2004 ± 0.00624	41.01 ± 0.887	59.23 ± 1.497	4.215 ± 0.0787	5.945
YAM:0.02Tb^3+^	0.2115 ± 0.00633	38.85 ± 0.789	56.47 ± 1.462	4.829 ± 0.0741	6.625
YAM:0.03Tb^3+^	0.2137 ± 0.00689	38.45 ± 0.734	53.68 ± 1.403	5.231 ± 0.0721	6.764
YAM:0.04Tb^3+^	0.2179 ± 0.00671	37.71 ± 0.912	51.02 ± 1.042	6.148 ± 0.0709	7.032
YAM:0.05Tb^3+^	0.2214 ± 0.00705	37.54 ± 0.733	48.25 ± 1.135	6.783 ± 0.0712	7.095

### 3.2 SEM and TEM examination

**Fig. 6:**
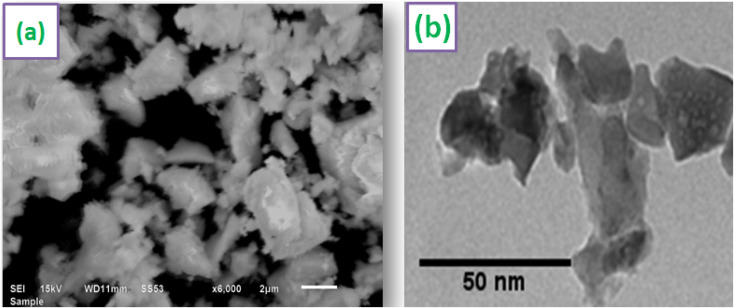
Micrographs (a) SEM (b) TEM of optimized Y_3.97_Al_2_O_9_:0.03Tb^3+^ sample.

SEM image of Y_3.97_Al_2_O_9_:0.03Tb^3+^ nanopowder is shown in Fig. 6(a). According to the SEM profile, particles in the optimized sample have a non-uniform shape and lie in micron-size. This might be as a result of the agglomeration caused by the combustion of material, which releases gas byproducts during the combustion. To comprehend the morphological characteristics (shape & size) of crystalline materials, TEM exploration has been used. TEM micrograph of materials that were re-heated at 800 °C is shown in Fig. 6(b). The shape of the particles found to be non-spherical and range in size from 20–55 nm. The small misdeeds in particle's shape and size were due to the uneven distribution of heat and mass during material ignition.^46^ The results of TEM study and the diffraction examinations are in close alliance.

### 3.3 FTIR study

Fig. S1† displays the FTIR spectral patterns of Y_3.97_Al_2_O_9_:0.03Tb^3+^ nanophosphors recorded in 400–4000 cm^−1^. Band at 3446 cm^−1^ equivalent to –OH unit of water suggested the presence of moisture content in the synthesized materials. A weak band at ∼2364 cm^−1^ is assigned to the stretching vibrations of nitrate (–NO_3_) unit.^47^ The vibrational bands at ∼442 and 568 cm^−1^ are demonstrates the Al–O bond.^48^ The bands present at 716 cm^−1^ and 791 cm^−1^ are assigned to Y–O bonds.^49^ The results of FTIR study fairly corroborate with the XRD analysis data.

### 3.4 EDX investigation

**Fig. 7:**
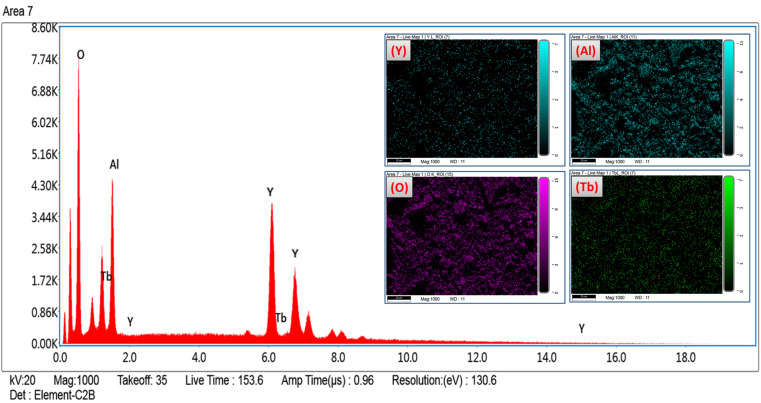
EDX spectral profile of optimized Y_3.97_Al_2_O_9_:0.03Tb^3+^ phosphors and inset represent the field view corresponding to each atoms present in Y_3.97_Al_2_O_9_:0.03Tb^3+^ sample.

The formation of pure Y_4_Al_2_O_9_:Tb^3+^ nanophosphor has been confirmed *via* EDX spectral profile. The EDX profile (Fig. 7) evinced various peaks attributed to the elements present in the prepared lattice. EDX pattern of nano-phosphors consist peaks exclusively of yttrium, aluminum, oxygen and terbium suggesting the nonexistence of any additional element in the lattice and formation of desired lattice in proper stoichiometry. Inset of Fig. 7 demonstrates the field view corresponding to the particular elements present in the synthesized optimum sample. The peculiar peaks of Tb^3+^ cation confirmed the uniform doping of former ion in the synthesized lattice. Table 4 evinces the EDX data (atomic and weight %) of Y_3.97_Al_2_O_9_:0.03Tb^3+^ nanophosphor. The outcomes of EDX specify the composition of homogeneous phosphor in desired stoichiometry with appropriate distribution of elements. These results are also found in accordance with structural and spectral data.

**Table 4:** Atomic & weight percentage of the optimized YAM:0.03Tb^3+^ phosphors

Element	Series	Weight (%)	Atomic (%)	Net intensity
Oxygen (O)	K-series	26.51	64.35	402.39
Aluminium (Al)	K-series	14.80	21.30	254.07
Yttrium (Y)	L-series	55.83	13.81	373.24
Terbium (Tb)	L-series	2.86	0.68	13.70

### 3.5 Optical absorption analysis

**Fig. 8:**
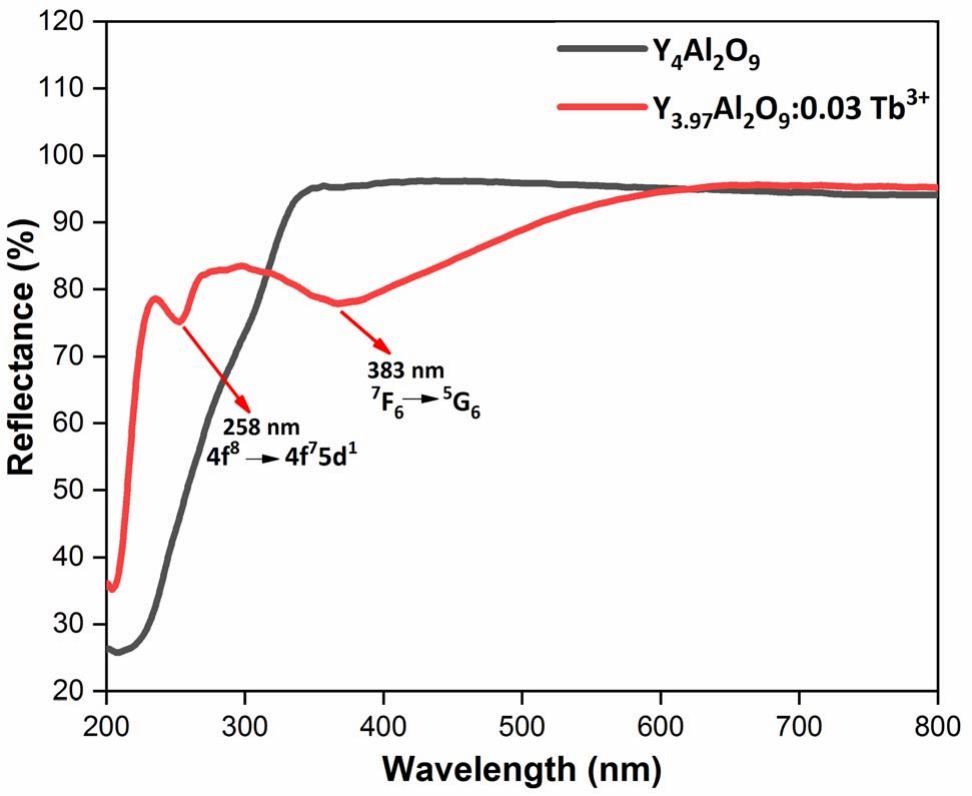
Diffusion reflectance spectral profiles of host and optimized Y_3.97_Al_2_O_9_:0.03Tb^3+^ nanophosphor.

Fig. 8 demonstrates the diffusion reflectance spectra (DRS) spectra measured between 200 and 800 nm wavelength range which was used to determine the optical characteristics and energy band gap of undoped Y_4_Al_2_O_9_ and optimized Y_3.97_Al_2_O_9_:0.03Tb^3+^ nanosamples. From the figure, it is clear that there exist characteristic transitions of Tb^3+^ ions located at 258 nm and 383 nm accredited with 4f^8^ → 4f^7^5d^1^ and ^7^F_6_ → ^5^G_6_ respectively. However, in the reflectance spectrum of host lattice no such peaks were observed. Additionally, the Kubelka–Munk function (eqn (4)) may be employed to define energy band gap of synthesized nanophosphors given as^50^4
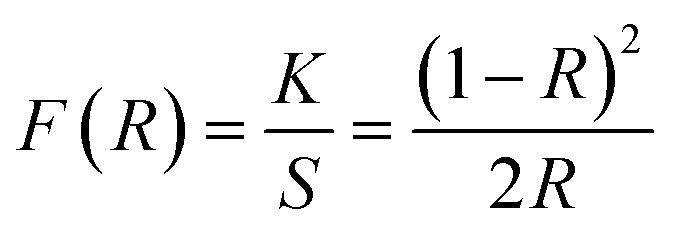
Here, *R* is calculated reflectance normalized with BaSO_4_ and *K* & *S* represent the absorption and scattering coefficients, respectively. Though, relationship between band gap (*E*_g_) and absorption-factor (*α*) of considered crystalline sample is determined through utilizing Tauc's relation (eqn (5))5
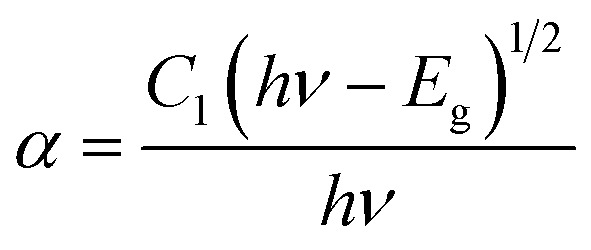
Here, *hν* stands for photon energy. Combining eqn (4) and (5) gives an evaluation of the band gap energy values, which is then expressed as^51^

6 [*F*(*R*)*hν*]^2^ = *C*_2_(*hν* − *E*_g_)

Here, the *hν* refers to the photon energy, *C*_2_ is a constant and 2 represent the direct band transition. The direct band gap values are estimated by drawing a tangent to each line that intersects the *x*-axis (energy) in Fig. S2(a) & (b),† which shows in the [*F*(*R*)*hν*]^2^*vs. hν* plot. The optical band gap values for host Y_4_Al_2_O_9_ and optimized nanosamples were calculated to be 5.10 eV and 5.67 eV respectively.

### 3.6 Photoluminescence analysis

#### 3.6.1 Excitation and emission spectrum of bulk material

**Fig. 9:**
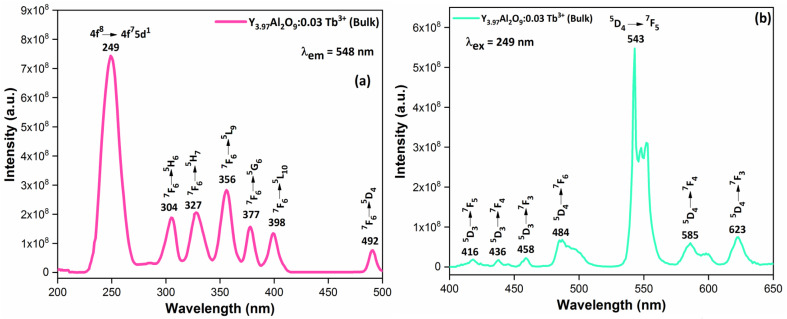
Photoluminescence spectrum (a) excitation and (b) emission of the Y_3.97_Al_2_O_9_:0.03Tb^3+^ bulk phosphors.

Fig. 9(a) demonstrates the excitation spectrum of Y_3.97_Al_2_O_9_:0.03Tb^3+^ bulk material. The excitation spectrum recorded at 548 nm, consists of one highly intense band at 249 nm from the 4f^8^ → 4f^7^5d^1^ transition of Tb^3+^ ion and some sharp peaks from the 4f^8^ → 4f^8^ transition of Tb^3+^ ions in the longer wavelength region located at 304, 327, 356, 377, 398 and 492 nm corresponding to the electronic transition from the ^7^F_6_ ground states to ^5^H_6_, ^5^H_7_, ^5^L_9_, ^5^G_6_, ^5^L_10_ and ^5^D_4_ respectively.^52^ When the bulk Y_3.97_Al_2_O_9_:0.03Tb^3+^ material was excited at a wavelength of 249 nm, the Tb^3+^ ion (4f^8^) would be raised to the higher ^4^f_7_5d^1^ level and would feed afterward to the ^5^D_3_ or ^5^D_4_ excited states. The PL spectra of bulk Y_3.97_Al_2_O_9_:0.03Tb^3+^ material reveals several emission peaks at 416, 436, 458, 484, 543, 585 and 623 nm, which are attributed to the electronic transitions ^5^D_3_ → ^7^F_5_, ^5^D_3_ → ^7^F_4_, ^5^D_3_ → ^7^F_3_, ^5^D_4_ → ^7^F_6_, ^5^D_4_ → ^7^F_5_, ^5^D_4_ → ^7^F_4_ and ^5^D_4_ → ^7^F_3_ respectively,^53^ as shown in Fig. 8(b). The emission spectrum lines can be separated in two groups. The blue emission group is from ^5^D_3_ → ^7^F_*J*_ (*J* = 5, 4 and 3) below 480 nm and the green emission group is ^5^D_4_ → ^7^F_*J*_ (*J* = 6, 5, 4 and 3) above 480 nm. From the Fig. 9, it is clear that the intensity of the excitation and emission spectra found to be high as compared to the optimized nanophosphors. The reason behind this high intensity is high temperature sintering (1100 °C) solid state reaction (SSR) method. Also, the excitation and emission spectra of bulk materials differ from the nanophosphors in respect of their shape and position of the peaks. Also, the loss of intensity in nanophosphor is due to crystalline defects and micro deformations acting as photoluminescence quenchers.^54^ Furthermore, it has been reported that surface –OH groups are efficient quenchers when phosphors are synthesized by wet synthesis methods.^55^

#### 3.6.2 Excitation and emission spectra of nanophosphors

**Fig. 10:**
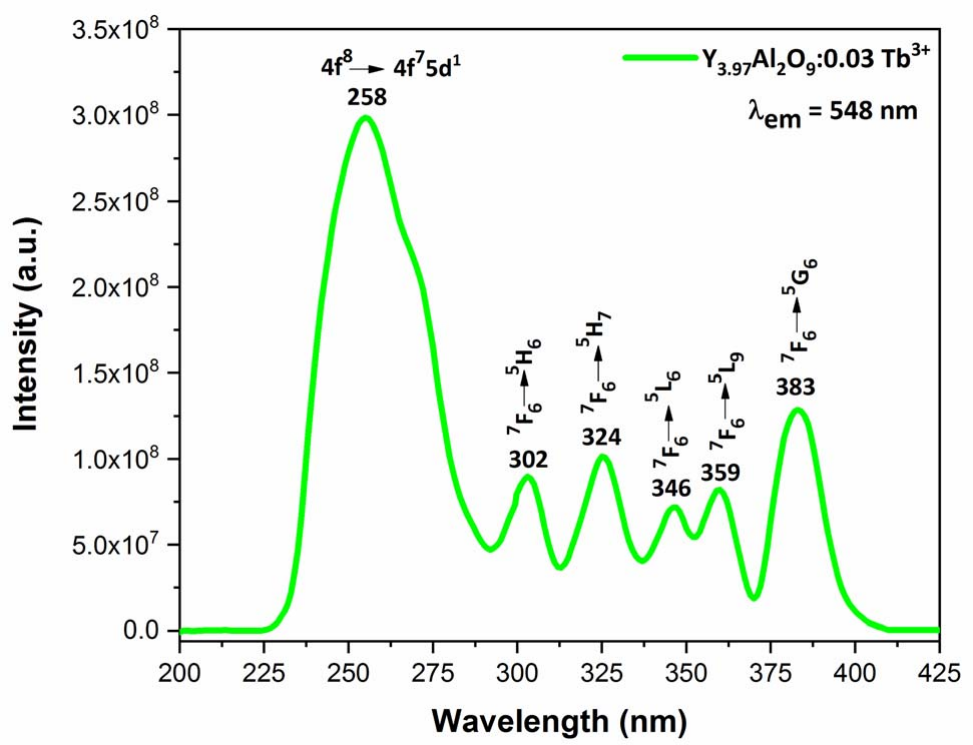
Excitation spectrum of Y_3.97_Al_2_O_9_:0.03Tb^3+^ nanophosphors monitored at 548 nm emission wavelength.

**Fig. 11:**
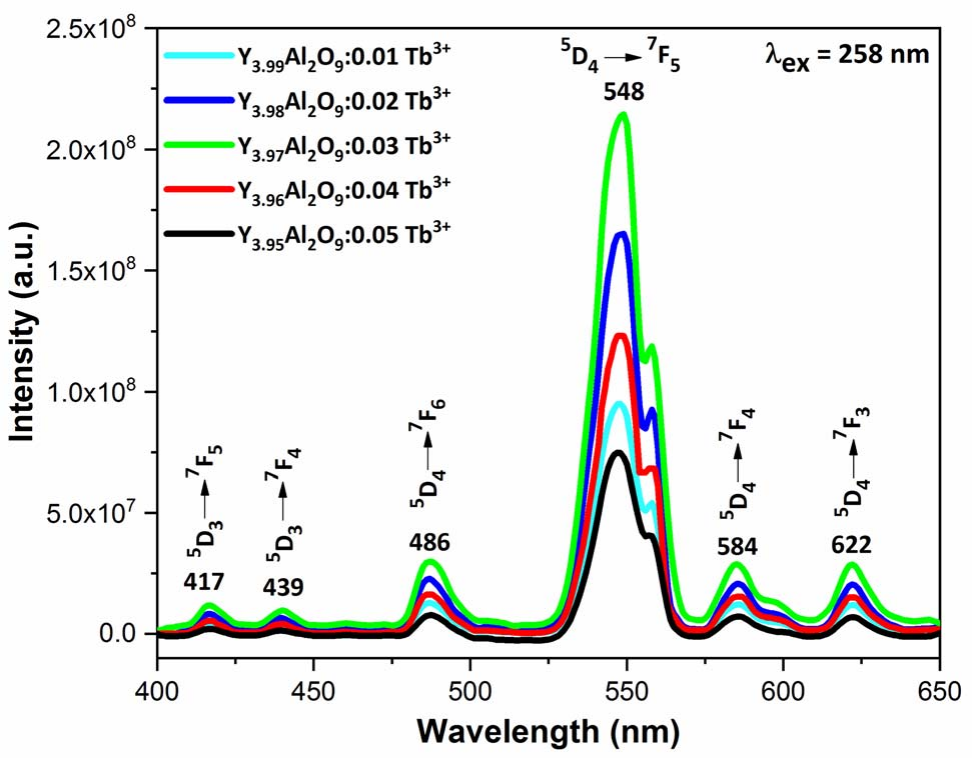
Emission spectral profiles of Y_4−*x*_Al_2_O_9_:*x*Tb^3+^ (*x* = 1–5 mol%) nanophosphors.

**Fig. 12:**
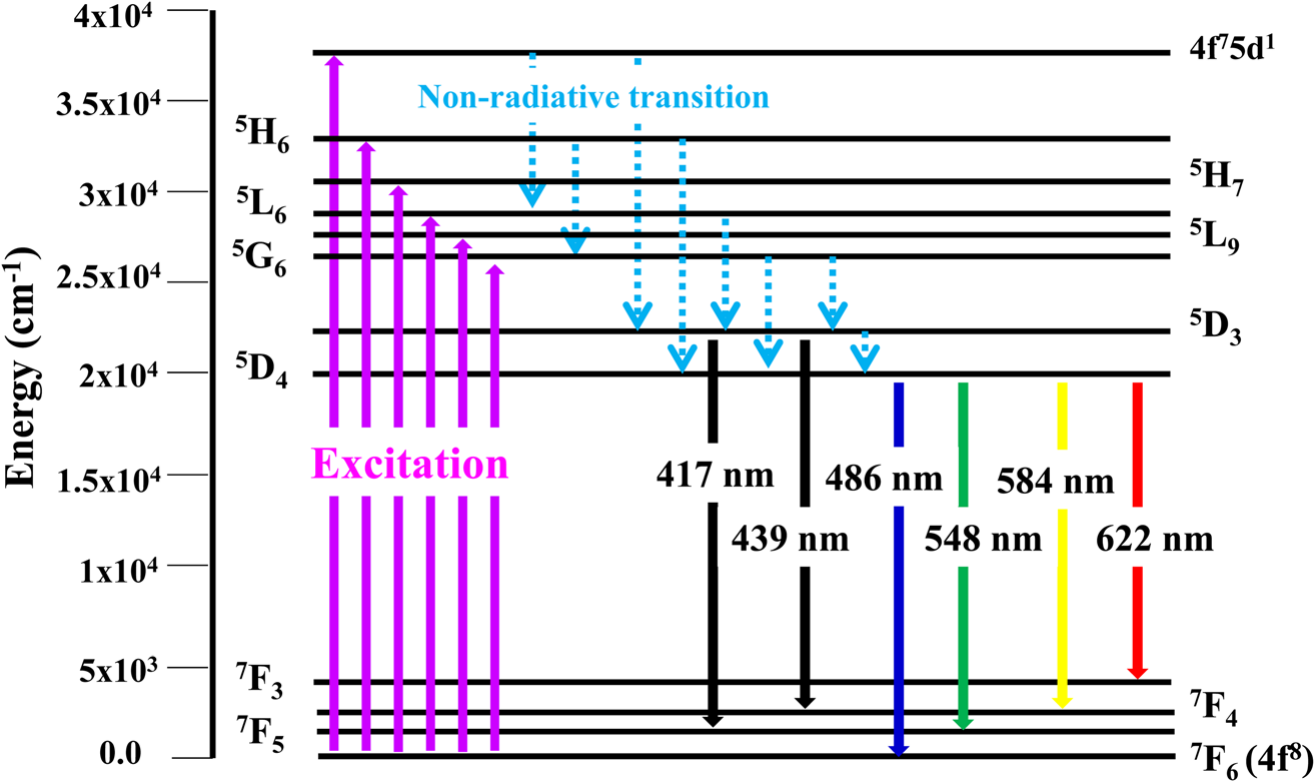
Different energy levels of Tb^3+^ ion in synthesized nanophosphors.

PLE behaviour of Y_3.97_Al_2_O_9_:0.03Tb^3+^ nanophosphor was revealed in Fig. 10. The excitation spectrum has been examined at Tb^3+^ emission of 548 nm, which mainly contains most intense and broad band at 258 nm derived from the 4f^8^ → 4f^7^5d^1^ transition of Tb^3+^ along with excitation bands at ∼302 nm, 324 nm, 346 nm, 359 nm and 383 nm with ^7^F_6_ → ^5^H_6_, ^7^F_6_ → ^5^H_7_, ^7^F_6_ → ^5^L_6_, ^7^F_6_ → ^5^L_9_ and ^7^F_6_ → ^5^G_6_ transitions, individually.^56^ Among all the excitation bands, most intense excitation 4f^8^ → 4f^7^5d^1^ transition located at 258 nm was used to monitor the PL spectra of synthesized nanocrystalline sample. In the present synthesized nanocrystalline materials, there are four emission centres (Y1/Tb1, Y2/Tb2, Y3/Tb3 and Y4/Tb4) with two different coordinating sites (Y2/Tb2 and Y4/Tb4 are in six coordinated octahedral environment and Y1/Tb1 and Y3/Tb3 are in seven coordination field) are present. The PL characteristic of all the synthesized nanosamples *viz.* Y_4−*x*_Al_2_O_9_:*x*Tb^3+^ (*x* = 1–5 mol%) was noted at 258 nm excitation, as displayed in Fig. 11. Normally, the luminous behaviour of Tb^3+^ is due to de-excitation from excited states (^5^D_3_ and ^5^D_4_) to the ^7^F_*J*=0–6_. Nanosamples get energized to the 4f^8^ state on absorbing photons of 258 nm wavelength and depopulated to the above mentioned excited states. Emission spectra entail different emissive peaks namely ^5^D_3_ → ^7^F_5_ (at 417 nm), ^5^D_3_ → ^7^F_4_ (at 439 nm), ^5^D_4_ → ^7^F_6_ (at 486 nm), ^5^D_4_ → ^7^F_5_ (at 548 nm), ^5^D_4_ → ^7^F_4_ (at 584 nm) and ^5^D_4_ → ^7^F_3_ (at 622 nm). PL spectra are divided into two regions which are known as blue region and green region.^57,58^ The obtained emission bands *i.e.* at 417 nm (^5^D_3_ → ^7^F_5_) and at 439 nm (^5^D_3_ → ^7^F_4_) are belongs to the blue region of the spectra while the transition ^5^D_4_ → ^7^F_*J*_ (*J* = 3, 4, 5 and 6) related to the green section of the spectra.^59^ Hyper-intense peak observed at 548 nm (^5^D_4_ → ^7^F_5_) was liable for green in synthesized nanosamples. Different energy levels of trivalent terbium ion are displayed in Fig. 12.

#### 3.6.3 Concentration quenching mechanism

To analyze the concentration effect of Tb^3+^ ion on the emission intensity of Y_4−*x*_Al_2_O_9_:*x*Tb^3+^ (*x* = 1–5 mol%) nanophosphor, samples of numerous Tb^3+^ ion content extending from 1 to 5 mol% have been fabricated. Fig. S3† demonstrates the relation of emission intensity with respect to the concentration of Tb^3+^ ion. This figure clearly revealed that initially the emission intensity increase and reached the maxima at 3 mol% Tb^3+^ ion, and then it decrease up to 5 mol% of Tb^3+^ ion concentration. This decrease in PL intensity at high concentration of Tb^3+^ was the result of concentration quenching. This concentration quenching effect can be understands by the Dexter energy transfer mechanism. Commonly, two main aspects are responsible for the energy transfer between the neighbouring activator ions, one is exchange interaction (for which critical distance must be less than 5 Å) and second is multipolar interaction (for which critical distance should be greater than 10 Å). For the estimation of the critical distance (*R*_c_), Blasse relation was used which is given by eqn (7) (ref. 60)7
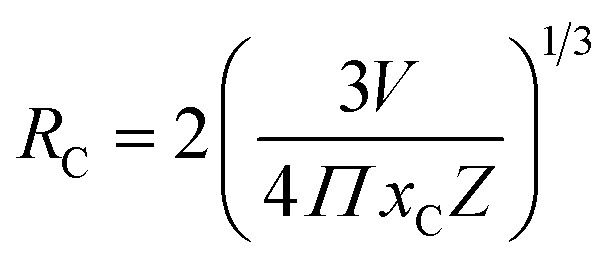
Where, *x*_C_ refers the critical concentration of Tb^3+^ ions, *V* denotes cell volume and *Z* stands for a number of cations in a single unit-cell. For optimized sample, volume = 828.72 Å^3^ and *Z* = 4 and the calculated *R*_C_ value is 23.63 Å which validates that energy transfer takes place *via* multipolar interactions. To further authenticate the mechanism of energy transfer in Y_4−*x*_Al_2_O_9_:*x*Tb^3+^ (*x* = 1–5 mol%) nanophosphors, Dexter's formula of multipolar interaction is used as follows eqn (8)8
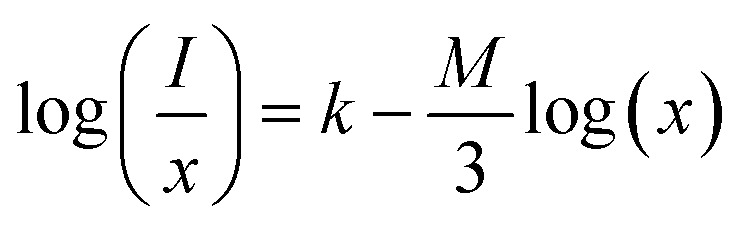


**Fig. 13:**
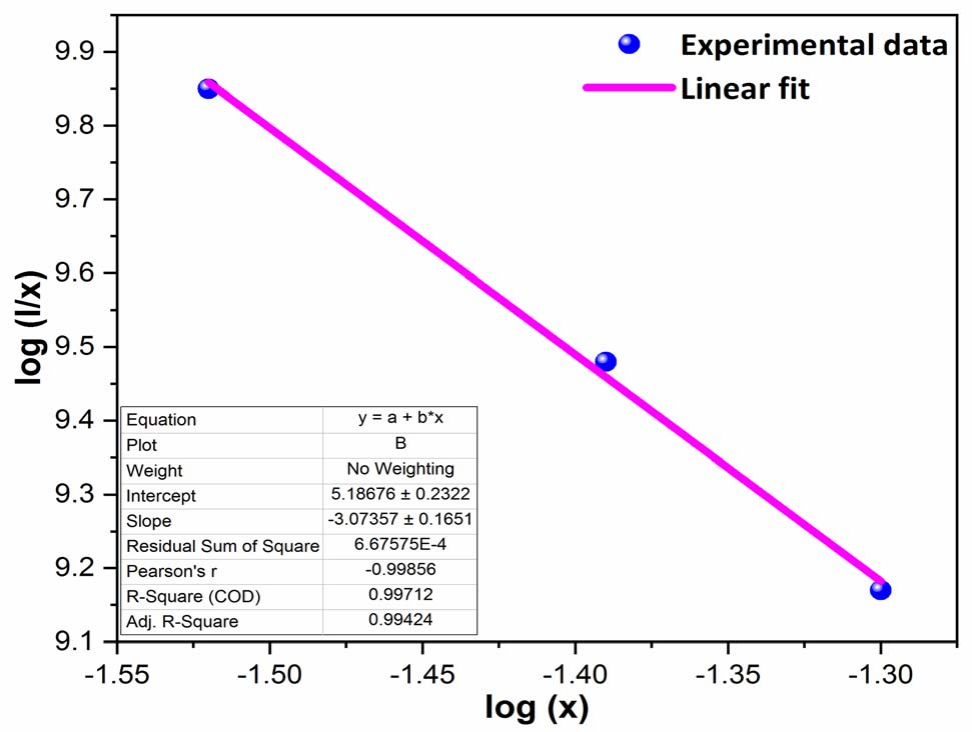
Linear fitted profile after quenching of concentration.

The multipolar-interaction comprises quadruple–quadruple (*M* = 10), dipole–quadruple (*M* = 8), dipole–dipole (*M* = 6) and migration of energy among nearest ions (*M* = 3).^61^ Correlation between log(*x*) *vs.* log(*I*/*x*) is obtained with linear fit graph, as exhibited in Fig. 13. The obtained slope is −3.07, resulted into *Q* value of 9.21. This result suggesting that quadruple–quadruple interactions are answerable for concentration quenching (CQ) in prepared nanosamples.

### 3.7 Luminescence lifetime and quantum efficiency

**Fig. 14:**
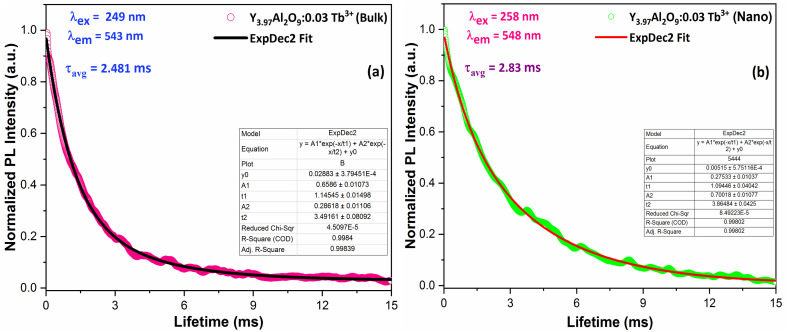
Lifetime decay graph of (a) bulk and (b) Y_3.97_Al_2_O_9_:0.03Tb^3+^ nanophosphor.

The decay lifetime of Y_3.97_Al_2_O_9_:0.03Tb^3+^ bulk and nanophosphor was monitored under an excitation on 249 nm (bulk, 4f^8^ → 4f^7^5d^1^) and 258 nm (nanophosphor, 4f^8^ → 4f^7^5d^1^) and emission at 543 nm (bulk, ^5^D_4_ → ^7^F_5_) and 548 nm (nanophosphor, ^5^D_4_ → ^7^F_5_) as demonstrates in Fig. 14(a) & (b). The decay curve is perfectly fitted through second order exponential method and the formula is given below

9 *I*_*t*_ = *I*_0_ + *A*_1_ exp(−*t*/*τ*_1_) + *A*_2_ exp(−*t*/*τ*_2_)

The above formulation incudes *A*_1_ and *A*_2_ which are the fitting parameters, *I*_0_ and *I*_*t*_ (intensity at *t* = 0 and *t* respectively). *τ*_1_ & *τ*_2_ are the exp components of decay lifetimes, individually. *τ*_avg_ can be measured by the relation (10) as^62^

10 *τ*_avg_ = (*A*_1_*t*_1_^2^ + *A*_2_*t*_2_^2^)/(*A*_1_*t*_1_ + *A*_2_*t*_2_)

The average lifetime values of Y_3.97_Al_2_O_9_:0.03Tb^3+^ bulk and nanophosphor 2.481 ms and 2.831 ms respectively. The average decay lifetimes (*τ*_avg_) for all synthesized doped nanosamples are shown in Table 5. As we can observe, decay times decrease as the doping amount of trivalent terbium ion increases. As content of incoming ions rises, then ions move in closer proximity to one another and quickly transfer energy, providing different decay-path with reduced decay-lifetime. Fig. S4† reveals that the *τ*_0_ value becomes 3.69 ms, obtained *via* Auzel's fitting by the use of eqn (11)11
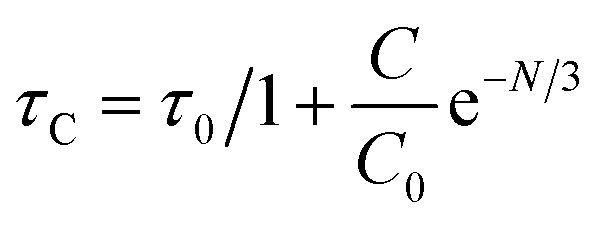
Where, *τ*_C_ represent decay lifetime, *C*_0_ denotes constant value and *N* are number of phonon. Additionally, value of internal quantum efficiency (*η*) of activated nanosamples is intended by the ratio of average lifetime and radiative lifetime (eqn (12)).^63^

12 *η* = *τ*_avg_/*τ*_0_

**Table 5:** Chromaticity parameters, lifetime values and quantum efficiency of the synthesized Y_4−*x*_Al_2_O_9_:*x*Tb^3+^ (*x* = 1–5 mol%) nanophosphors

Sample	(*x*, *y*)	(*u*′, *v*′)	CCT (K)	CP (%)	*τ*_avg_	*η*_int_ (%)	*η*_e*x*t_ (%)
1 mol% Tb^3+^	0.274, 0.575	0.116, 0.551	6619.29	90.52	3.36	91.05	68.14
2 mol% Tb^3+^	0.234, 0.550	0.102, 0.521	7782.24	87.26	3.12	84.56	57.88
3 mol% Tb^3+^	0.254, 0.563	0.113, 0.543	8746.29	89.01	2.83	76.69	50.19
4 mol% Tb^3+^	0.314, 0.541	0.138, 0.549	7229.56	76.29	2.69	72.89	46.27
5 mol% Tb^3+^	0.224, 0.526	0.102, 0.536	8046.76	80.48	2.47	66.93	38.15

Internal quantum efficiency (*η*) of selected nanophosphors is assessed to be 91.05, 84.56, 76.69, 72.89 and 66.93% for *x* = 1, 2, 3, 4 and 5 mol% respectively. These values suggest that the quantum efficiency continuous decreases with trivalent terbium concentration. Photoluminescence quantum efficiency is termed as the ratio of emissive photons to the absorbed photons. The external luminescence quantum yield of synthesized Y_4−*x*_Al_2_O_9_:*x*Tb^3+^ (*x* = 1–5 mol%) nanophosphors was measured on a Fluorolog-3 Horiba Jobin Yvon equipped with a 150 W pulsed xenon lamp. External quantum efficiency measurements were done at room temperature at an excitation wavelength of 258 nm with a slit width of 1 nm for excitation and 1 nm for emission.

### 3.8 Colorimetric investigation

**Fig. 15:**
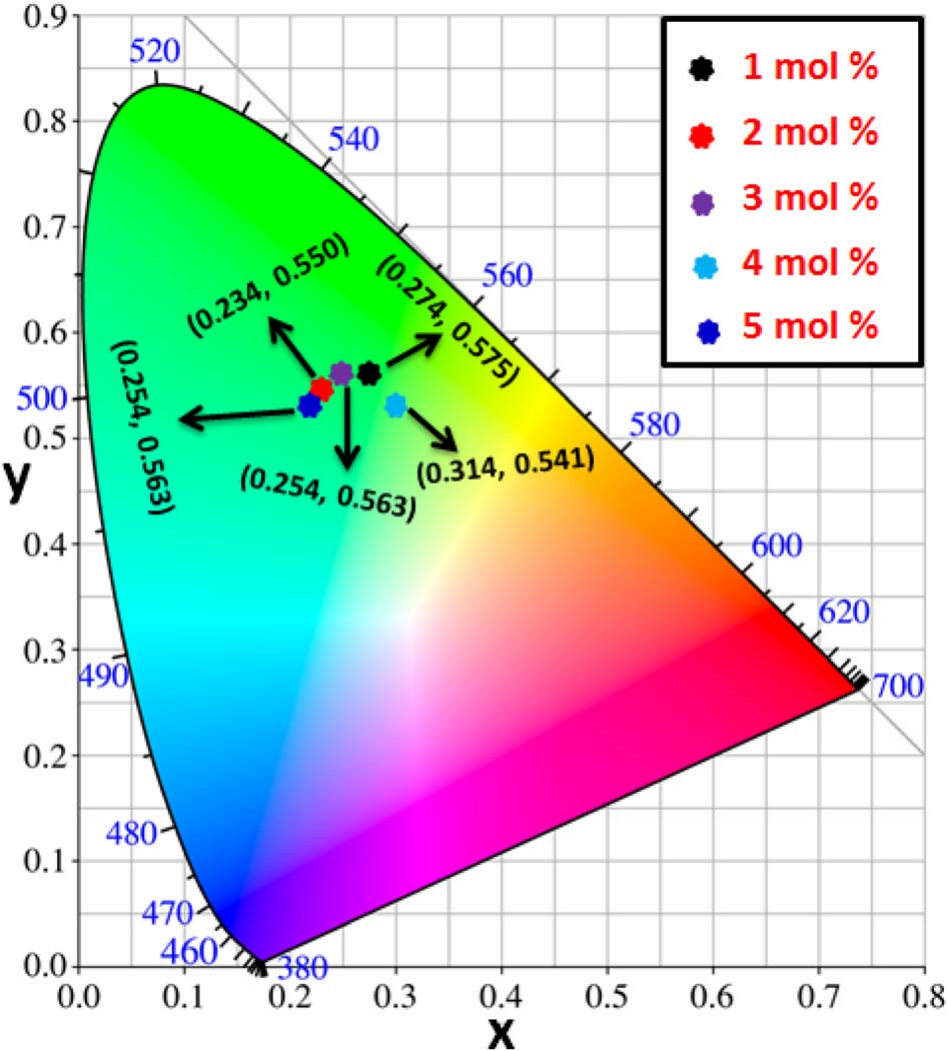
CIE profile of the synthesized Y_4−*x*_Al_2_O_9_:*x*Tb^3+^ (*x* = 1–5 mol%) nanophosphors.

Using CIE chromaticity diagram, the colors of Y_4−*x*_Al_2_O_9_:*x*Tb^3+^ (*x* = 1–5 mol%) nanophosphors display applications is determined. Table 5 depicts the values of the chromaticity points that were computed from PL spectra (at 258 nm). According to Tb^3+^ ion concentration, the CIE coordinates are located in the green area as represented in the CIE diagram as revealed in Fig. 15. These results demonstrate the use of Y_4−*x*_Al_2_O_9_:*x*Tb^3+^ (*x* = 1–5 mol%) nanophosphors as a green component for display applications at various Tb^3+^ concentrations. Also, these coordinates approach to the Standard European Broadcasting Union of green coordinates. Typically, the CCT value identifies the type of light, such as warm or cold. Color temperature is calculated using the McCamy relation (13) (ref. 64 and 65)

13 CCT = −437*n*^3^ + 3601*n*^2^ − 6861*n* + 5514.31

Here, *n* = (*x* − *x*_e_)/(*y* − *y*_e_), *x*_e_ & *y*_e_ are the chroma epicenters with value 0.332 & 0.186 respectively. The other coordinates were assessed from (*x* & *y*) through eqn (14).14
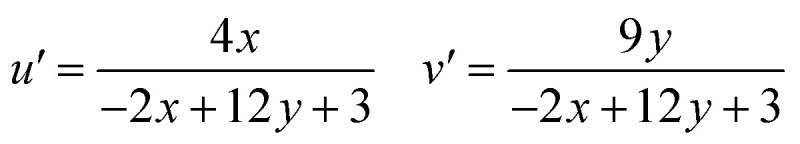


**Fig. 16:**
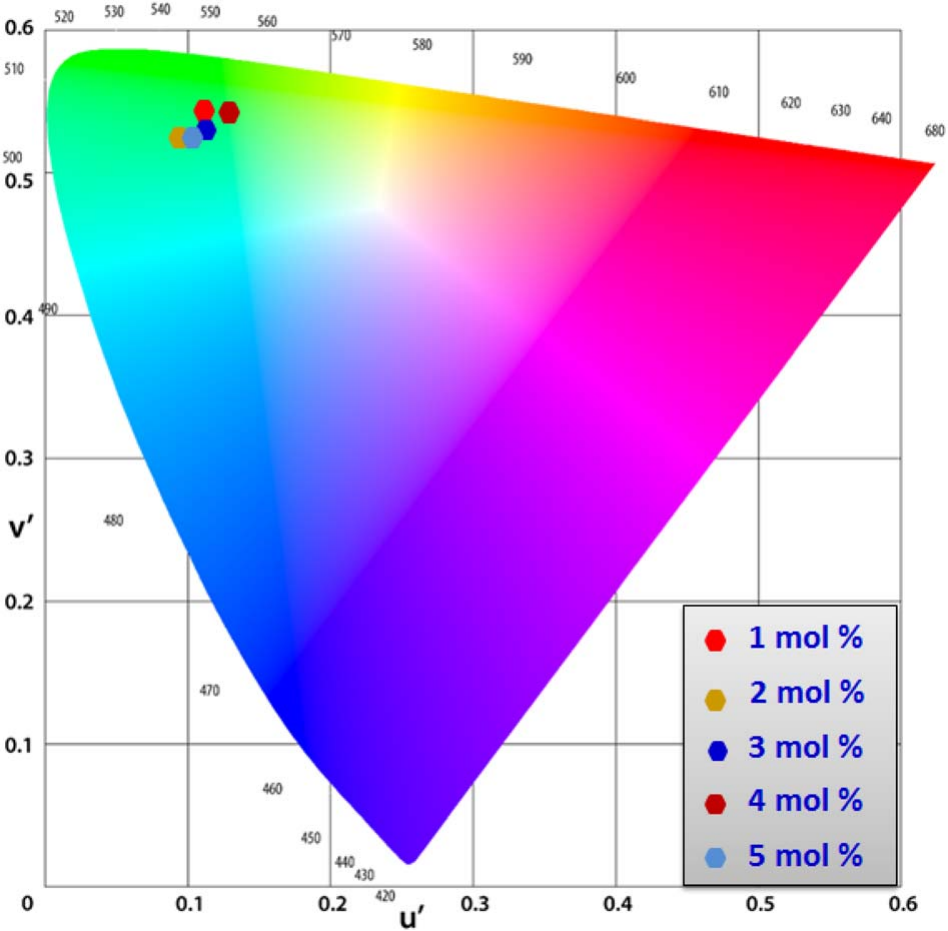
CCT value of Y_4−*x*_Al_2_O_9_:*x*Tb^3+^ (*x* = 1–5 mol%) nanophosphors.

Fig. 16 clarifies color temperature values of prepared phosphors. All developed nanosamples revealed CCT values between 6500 and 9000 K, demonstrating that the light is produced as cold source. The color purity of Y_4−*x*_Al_2_O_9_:*x*Tb^3+^ (*x* = 1–5 mol%) nanophosphors could be measured by eqn (15).^66,67^15
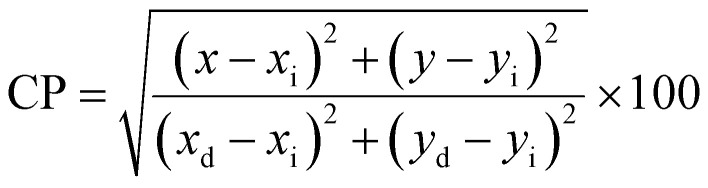
Here, (*x*_d_, *y*_d_) and (*x*_i_, *y*_i_) are the dominant and illuminated points separately. The color parameters for the chosen nanophosphors are listed in Table 5. The obtained Y_4−*x*_Al_2_O_9_:*x*Tb^3+^ (*x* = 1–5 mol%) nanophosphor results can be viewed as a promising candidate for display applications and other future lighting foundations.

## 4 Conclusions

The current study concludes that less time consuming, urea assisted gel-combustion synthetic approach was used to synthesize the powdered samples of cool green color emanating Y_4−*x*_Al_2_O_9_:*x*Tb^3+^ (*x* = 1–5 mol%) nanocrystalline materials. All cell characteristics determined *via* XRD aided Rietveld refinement technique which is in excellent agreement with those found in the literature, and the addition of Tb^3+^ had no discernible impact on the YAM phase structure.

The TEM observation of powdered sample revealed particles with a diameter of less than 50 nm with agglomeration caused by coalescence, resemble with size predicted by Scherrer's equation utilizing XRD patterns. The synthesized nanomaterials found to be non-conducting in nature validates by their optical band gap values. The emission spectra recorded at 258 nm contains dominating band at 548 nm with ^5^D_4_ → ^7^F_5_, responsible for green color in synthesized nanomaterials. The quadruple–quadruple interactions are accountable for the quenching of concentration in the prepared phosphors. The computed luminescent characteristics (quantum efficiency, CIE and CCT) for all nanosamples explore as potential candidates for the solid state lighting applications.

## Data availability

Data will be made available on request.

## Author contributions

Pawan Kumar: data curation, writing – original draft, investigation, methodology; Devender Singh: writing – review & editing, resources, supervision; Isha Gupta: validation; Sitender Singh: formal analysis, project administration; Simran Nehra: software; Ramesh Kumar: visualization.

## Conflicts of interest

The authors declare that they have no known competing financial interests or personal relationships that could have appeared to influence the work reported in this paper.

## Supplementary Material

RA-013-D3RA00735A-s001
