# Supplementary material for: Combustion derived single phase Y4Al2O9:Tb3+ nanophosphor: crystal chemistry and optical analysis for solid state lighting applications
Source: RSC Adv. 2023 Mar 8;13(12):7752–65. doi: 10.1039/d3ra00735a (PMC9993242; doi:10.1039/d3ra00735a)
Supplement: RA-013-D3RA00735A-s001 [file RA-013-D3RA00735A-s001.pdf]

Electronic Supplementary Material (ESI) for RSC Advances.
This journal is © The Royal Society of Chemistry 2023

Combustion derived single phase $Y_4Al_2O_9:Tb^{3+}$ nanophosphor: Crystal chemistry and optical analysis for solid state lighting applications

Pawan Kumar[a], Devender Singh[a*], Isha Gupta[a], Sitender Singh[a], Simran Nehra[b] and Ramesh Kumar[c]

[a]*Department of Chemistry, Maharshi Dayanand University, Rohtak-124001, India.*
[b]*CSIR-National Physical Laboratory, Dr. K. S. Krishnan Marg, New Delhi-110012 India.*
[c]*Department of Chemistry, Kurukshetra University Kurukshetra-136119, Haryana, India.*

Electronic Supporting Information

Table S1. Type of bond and interionic bond distance (Å) of the optimized nanophosphor.

Bond Type	Bond Distance (Å)	Bond Type	Bond Distance (Å)
Al(5)–O(8)	1.7358	Tb2/Y(2)–O(9)	2.2547
Al(5)–O(9)	1.7264	Tb2/Y(2)–O(10)	2.3604
Al(5)–O(10)	1.7378	Tb2/Y(2)–O(13)	2.3251
Al(5)–O(11)	1.7431	Tb2/Y (2)–O(14)	2.2802
Al(6)–O(7)	1.7696	Tb3/Y(3)–O(8)	2.3014
Al(6)–O(11)	1.7998	Tb3/Y(3)–O(10)	2.2304
Al(6)–O(12)	1.7368	Tb3/Y(3)–O(11)	2.3327
Al(6)–O(13)	1.7614	Tb3/Y(3)–O(12)	2.2893
Tb1/Y(1)–O(7)	2.4731	Tb3/Y(3)–O(14)	2.2803
Tb1/Y(1)–O(8)	2.4862	Tb3/Y(3)–O(15)	2.2762
Tb1/Y(1)–O(9)	2.2827	Tb3/Y(3)–O(15)	2.2909
Tb1/Y(1)–O(11)	2.3946	Tb4/Y(4)–O(7)	2.3157
Tb1/Y(1)–O(12)	2.2690	Tb4/Y(4)–O(10)	2.2303
Tb1/Y(1)–O(13)	2.2958	Tb4/Y(4)–O(12)	2.2892
Tb1/Y(1)–O(15)	2.1999	Tb4/Y(4)–O(14)	2.3090
Tb2/Y(2)–O(7)	2.2170	Tb4/Y(4)–O(14)	2.3712
Tb2/Y(2)–O(8)	2.2707	Tb4/Y(4)–O(15)	2.3034

Table S2. Refined atomic characteristics of the optimized_$Y_{3.97}Al_2O_9$:0.03Tb^{3+} nanophosphors.

Atom	O.S	Wyck	Site	x/a	y/b	z/c	U[Å^2]
Y1	+3	4e	0.9716	0.52210	0.10950	0.78520	0.0194
Y2	+3	4e	0.9485	0.02430	0.09590	0.80550	0.0191
Y3	+3	4e	0.9736	0.33930	0.12740	0.43690	0.0192
Y4	+3	4e	0.9512	0.83770	0.12140	0.41630	0.0194
Al5	+3	4e	1.0000	0.21560	0.18010	0.12770	0.0200
Al6	+3	4e	1.0000	0.65550	0.17740	0.11270	0.0200
O7	-2	4e	1.0000	0.78500	0.23530	0.74970	0.0200
O8	-2	4e	1.0000	0.23100	0.23690	0.76490	0.0200
O9	-2	4e	1.0000	0.21300	0.01840	0.15880	0.0200
O10	-2	4e	1.0000	0.07210	0.23490	0.98040	0.0200
O11	-2	4e	1.0000	0.42900	0.23940	0.11450	0.0200
O12	-2	4e	1.0000	0.63930	0.23200	0.96180	0.0200
O13	-2	4e	1.0000	0.69400	0.01700	0.16200	0.0200
O14	-2	4e	1.0000	0.08000	-0.00400	0.3800	0.0200
O15	-2	4e	1.0000	0.56300	0.00520	0.39000	0.0200
Tb1	+3	4e	0.0284	0.23690	0.21160	0.07200	0.1487
Tb2	+3	4e	0.0515	0.21560	0.07484	0.21860	0.1265
Tb3	+3	4e	0.0264	0.39120	0.21540	0.07824	0.1124
Tb4	+3	4e	0.0488	0.21540	0.06480	0.21470	0.1468

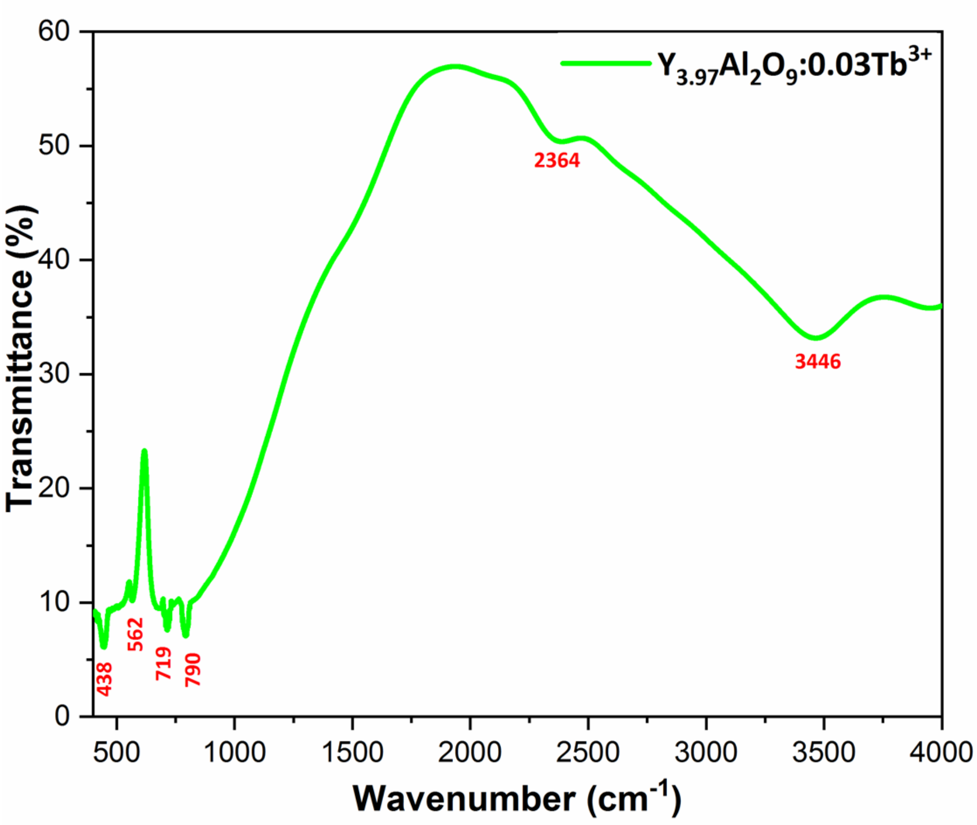

Fig. S1 FTIR spectral profile of $Y_{3.97}Al_2O_9$:$0.03Tb^{3+}$ nanosample.

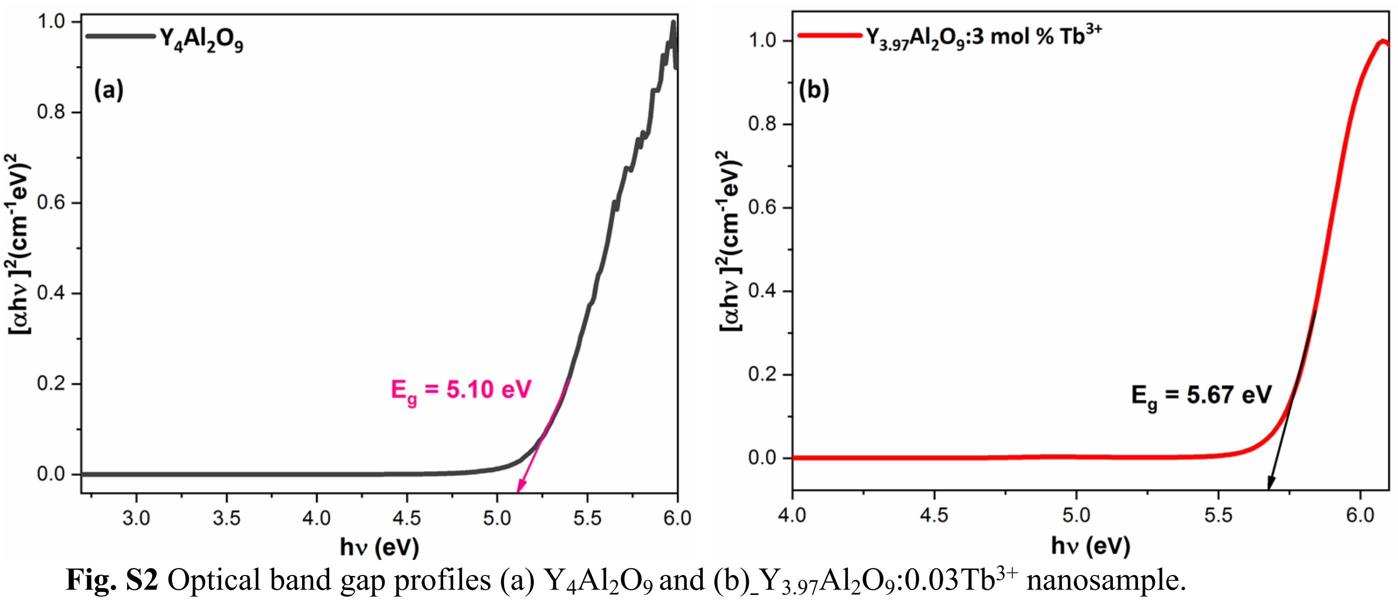

Fig. S2 Optical band gap profiles (a) $Y_4Al_2O_9$ and (b) $Y_{3.97}Al_2O_9$:$0.03Tb^{3+}$ nanosample.

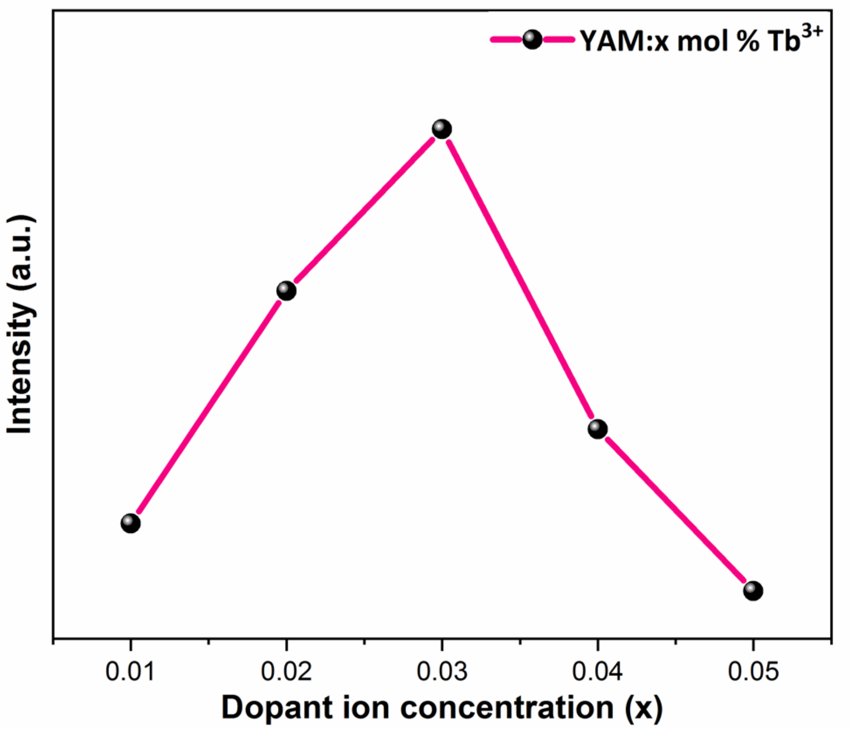

Fig. S3 Concentration quenching graph of_$Y_{4-x}Al_2O_9$:xTb^{3+} (x=1-5 mole %) nanophosphors.

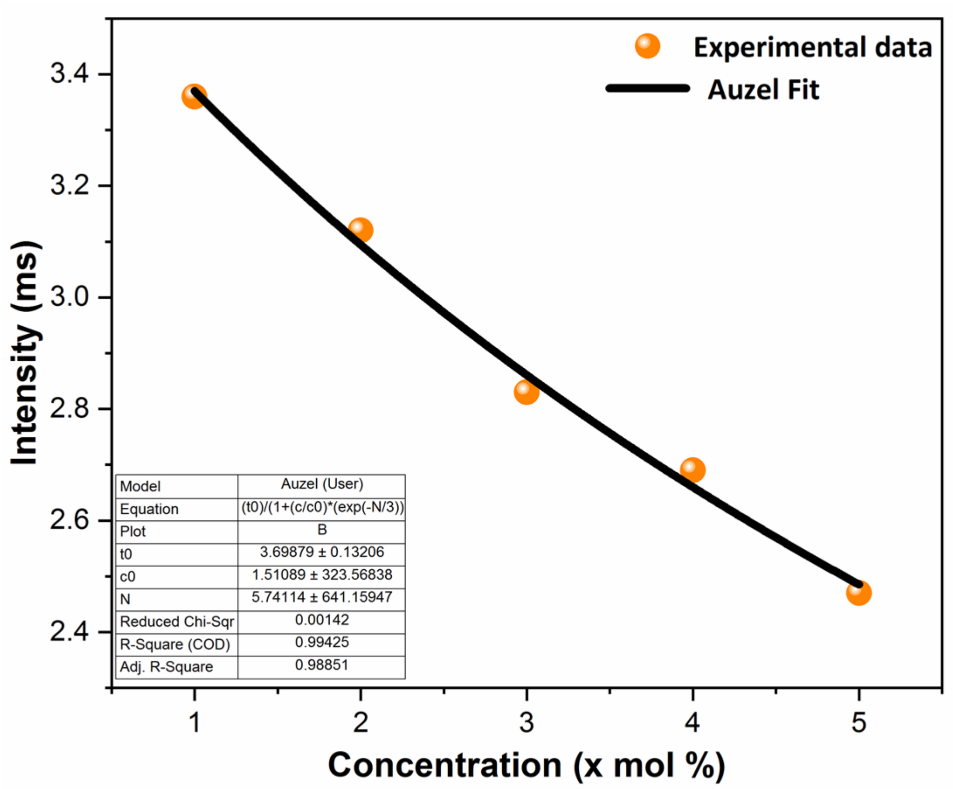

Fig. S4 Relation between decay time and dopant concentration (Auzel's fitting method).
